# *Chabertia erschowi* (Nematoda) is a distinct species based on nuclear ribosomal DNA sequences and mitochondrial DNA sequences

**DOI:** 10.1186/1756-3305-7-44

**Published:** 2014-01-22

**Authors:** Guo-Hua Liu, Lei Zhao, Hui-Qun Song, Guang-Hui Zhao, Jin-Zhong Cai, Quan Zhao, Xing-Quan Zhu

**Affiliations:** 1 State Key Laboratory of Veterinary Etiological Biology, Key Laboratory of Veterinary Parasitology of Gansu Province, Lanzhou Veterinary Research Institute, Chinese Academy of Agricultural Sciences, Lanzhou, Gansu Province 730046, PR China; 2 College of Animal Science and Technology, Jilin Agricultural University, Changchun, Jilin Province 130118, PR China; 3 College of Veterinary Medicine, Hunan Agricultural University, Changsha, Hunan Province 410128, PR China; 4 College of Veterinary Medicine, Northwest A&F University, Yangling, Shannxi Province 712100, PR China; 5 Qinghai Academy of Animal Science and Veterinary Medicine, Xining, Qinghai Province, PR China

**Keywords:** *Chabertia* spp, Nuclear ribosomal DNA, Internal transcribed spacer (ITS), Mitochondrial DNA, Phylogenetic analysis

## Abstract

**Background:**

Gastrointestinal nematodes of livestock have major socio-economic importance worldwide. In small ruminants, *Chabertia* spp. are responsible for economic losses to the livestock industries globally. Although much attention has given us insights into epidemiology, diagnosis, treatment and control of this parasite, over the years, only one species (*C. ovina*) has been accepted to infect small ruminants, and it is not clear whether *C. erschowi* is valid as a separate species.

**Methods:**

The first and second internal transcribed spacers (ITS-1 and ITS-2) regions of nuclear ribosomal DNA (rDNA) and the complete mitochondrial (mt) genomes of *C. ovina* and *C. erschowi* were amplified and then sequenced. Phylogenetic re-construction of 15 Strongylida species (including *C. erschowi*) was carried out using Bayesian inference (BI) based on concatenated amino acid sequence datasets.

**Results:**

The ITS rDNA sequences of *C. ovina* China isolates and *C. erschowi* samples were 852–854 bp and 862 -866 bp in length, respectively. The mt genome sequence of *C. erschowi* was 13,705 bp in length, which is 12 bp shorter than that of *C. ovina* China isolate. The sequence difference between the entire mt genome of *C. ovina* China isolate and that of *C. erschowi* was 15.33%. In addition, sequence comparison of the most conserved mt small subunit ribosomal (*rrn*S) and the least conserved *nad*2 genes among multiple individual nematodes revealed substantial nucleotide differences between these two species but limited sequence variation within each species.

**Conclusions:**

The mtDNA and rDNA datasets provide robust genetic evidence that *C. erschowi* is a valid strongylid nematode species. The mtDNA and rDNA datasets presented in the present study provide useful novel markers for further studies of the taxonomy and systematics of the *Chabertia* species from different hosts and geographical regions.

## Background

The phylum Nematoda includes many parasites that threaten the health of plants, animals and humans on a global scale. The soil-transmitted helminthes (including roundworms, whipworms and hookworms) are estimated to infect almost one sixth of all humans, and more than a billion people are infected with at least one species [1]. *Chabertia* spp. are common gastrointestinal nematodes, causing significant economic losses to the livestock industries worldwide, due to poor productivity, failure to thrive and control costs [2-6]. In spite of the high prevalence of *Chabertia* reported in small ruminants [7], it is not clear whether the small ruminants harbour one or more than one species. Based on morphological features (e.g., cervical groove and cephalic vesicle) of adult worms, various *Chabertia* species have been described in sheep and goats in China, including *C. ovina*, *C. rishati*, *C. bovis*, *C. erschowi*, *C. gaohanensis* sp. nov and *C. shaanxiensis* sp. nov [8-10]. However, to date, only *Chabertia ovina* is well recognized as taxonomically valid [11,12]. Obviously, the identification and distinction of *Chabertia* to species using morphological criteria alone is not reliable. Therefore, there is an urgent need for suitable molecular approaches to accurately identify and distinguish closely-related *Chabertia* species from different hosts and regions.

Molecular tools, using genetic markers in mitochondrial (mt) genomes and the internal transcribed spacer (ITS) regions of nuclear ribosomal DNA (rDNA), have been used effectively to identify and differentiate parasites of different groups [13-16]. For nematodes, recent studies showed that mt genomes are useful genetic markers for the identification and differentiation of closely-related species [17,18]*.* In addition, employing ITS rDNA sequences, recent studies also demonstrated that *Haemonchus placei* and *H. contortus* are distinct species [19]; *Trichuris suis* and *T. trichiura* are different nematode species [20,21].

Using a long-range PCR-coupled sequencing approach [22], the objectives of the present study were (i) to characterize the ITS rDNA and mt genomes of *C. ovina* and *C. erschowi* from goat and yak in China, (ii) to compare these ITS sequences and mt genome sequences, and (iii) to test the hypothesis that *C. erschowi* is a valid species in phylogenetic analyses of these sequence data.

## Methods

### Parasites and isolation of total genomic DNA

Adult specimens of *C. ovina* (n = 6, coded CHO1-CHO6) and *C. erschowi* (n = 9, coded CHE1-CHE9) were collected, *post-mortem,* from the large intestine of a goat and a yak in Shaanxi and Qinghai Provinces, China, respectively, and were washed in physiological saline, identified morphologically [8,10], fixed in 70% (v/v) ethanol and stored at -20°C until use. Total genomic DNA was isolated separately from 15 individual worms using an established method [23].

### Long-range PCR-based sequencing of mt genome

To obtain some mt sequence data for primer design, we PCR-amplified regions of *C. erschowi* of *cox*1 gene by using a (relatively) conserved primer pair JB3-JB4.5 [24], *rrn*L gene was amplified using the designed primers *rrn*LF (forward; 5′-GAGCCTGTATTGGGTTCCAGTATGA-3′) and *rrn*LR (reverse; 5′-AACTTTTTTTGATTTTCCTTTCGTA-3′), *nad*1 gene was amplified using the designed primers *nad*1F (forward; 5′-GAGCGTCATTTGTTGGGAAG-3′) and *nad*1R (reverse; 5′-CCCCTTCAGCAAAATCAAAC-3′), *cyt*b gene was amplified using the designed primers *cytb*F (forward; 5′-GGTACCTTTTTGGCTTTTTATTATA-3′) and *cyt*bR (reverse; 5′-ATATGAACAGGGCTTATTATAGGAT-3′) based on sequences conserved between *Oesophagostomum dentatum* and *C. ovina* Australia isolate. The amplicons were sequenced in both directions using BigDye terminator v.3.1, ABI PRISM 3730. We then designed primers (Table 1) to regions within *cox*1, *rrn*L, *nad*1 and *cyt*b and amplified from *C. ovina* (coded CHO1) in four overlapping fragments: *cox*1*-rrn*L, *rrn*L-*nad*1, *nad*1-*cyt*b and *cyt*b-*cox*1. Then we designed primers (Table 1) to regions within *cox*1, *rrn*L, *nad*5, *nad*1, *nad*2 and *cyt*b and amplified from *C. erschowi* (coded CHE1) in six overlapping fragments: *cox*1*- rrn*L, *rrn*L-*nad*5, *nad*5-*nad*1, *nad*1-*nad*2, *nad*2-*cyt*b and *cyt*b-*cox*1. The cycling conditions used were 92°C for 2 min (initial denaturation), then 92°C/10 s (denaturation), 50 -58°C (*C. erschowi*) or 56 -65°C (*C. ovina*)/30 s (annealing), and 60°C/10 min (extension) for 10 cycles, followed by 92°C for 2 min, then 92°C/10 s, 50 -58°C (*C. erschowi*) or 56 -65°C (*C. ovina*)/30 s, and 60°C/10 min for 20 cycles, with a cycle elongation of 10 s for each cycle and a final extension at 60°C/10 min. Each amplicon, which represented a single band in a 1.0% (w/v) agarose gel, following electrophoresis and ethidium-bromide staining, was column-purified and then sequenced using a primer walking strategy [22].

**Table 1:** **Sequences of primers used to amplify mitochondrial DNA regions from *Chabertia erschowi *** **and *Chabertia ovina *** **from China**

**Primer**	**Sequence (5′ to 3′)**
For *rrn*S	
CHOF	TCGTTTAGTGGGTATGTGTGGTTCT (for *C. ovina*)
CHOR	GCCTACTCCCTAACAAATGACGCTC (for *C. ovina*)
CHEF	GTGGTTTTTAGGTTAGGGTTGAGTG (for *C. erschowi*)
CHER	ACGCTCATACAAAGTAATAAACGCA (for *C. erschowi*)
For *nad*2	
CHOF	TTTGTGG(C\T)TAAGAGTGTT(G\A)GCTATT (for *C. ovina*)
CHOR	GAGCCGTAATCAAACATAGTAAATC (for *C. ovina*)
CHEF	TTTGTGG(C\T)TAAGAGTGTT(G\A)GCTATT (for *C. erschowi*)
CHER	ACCGTAATCAAACATAGTAAAATCT (for *C. erschowi*)
For *C. ovina*	
COF	TGGTTGTGTGGTTTGGGCTCAT
rrnLR	ATGTCCTCACGCTAAGACTGCC
rrnLF	AGTTTGCTTCTGCCCAGTGA
ND5R	ACCGTAACCTCGCCCATCCTG
ND5F	ACGGCGTTAGTGGAGGAGGA
ND1R	CCACTAACCAACTCCCTTTCACCC
ND1F	ATTGGTGCTTTGCGGGCCAGT
ND2R	CCATAAACCTTTAAAACCTCCC
ND2F	TTGTTGGTTGGGAGACTATG
CYR	AAAGGGTCCTCAACCAAACA
CYF	CCTGTTTGGGGACCTTCTATTG
COR	CCGCAGTAAAATAAGCACGAGA
For *C. erschowi*	
COF	ACCGACGGCTTATGGAAT
rrnLR	AGTGCAACCCAACATTATACCCT
rrnLF	TAAAGTTTGCTTCTGCCCAGTGATA
ND1R	ATAATAGCCAACAAAAGCACCGACA
ND1F	CTTGTCGGTGCTTTGCG
CYR	CCGCCTCAATAAACATCTC
CYF	TGGTCCAGATTATTGAAGG
COR	TTACCCGTCAAATACAAAGT

### Sequencing of ITS rDNA and mt *rrn*S and *nad*2

The full ITS rDNA region including primer flanking 18S and 28S rDNA sequences was PCR-amplified from individual DNA samples using universal primers NC5 (forward; 5′-GTAGGTGAACCTGCGGAAGGATCATT-3′) and NC2 (reverse; 5′-TTAGTTTCTTTTCCTCCGCT-3′) described previously [25]. The primers *rrn*SF and *rrn*SR (Table 1) designed to conserved mt genome sequences within the *rrn*S gene were employed for PCR amplification and subsequent sequencing of this complete gene (~ 700 bp) from multiple individuals of *Chabertia* spp. The primers *nad*2F and *nad*2R (Table 1) designed to conserved mt genome sequences within the *nad*2 gene were employed for PCR amplification and subsequent sequencing of this complete gene (~ 900 bp) from multiple individuals of *Chabertia* spp..

### Sequence analyses

Sequences were assembled manually and aligned against the complete mt genome sequences of *C. ovina* Australia isolate [26] using the computer program Clustal X 1.83 [27] to infer gene boundaries. Translation initiation and termination codons were identified based on comparison with that of *C. ovina* Australia isolate [26]. The secondary structures of 22 tRNA genes were predicted using tRNAscan-SE [28] and/or manual adjustment [29], and rRNA genes were identified by comparison with that of *C. ovina* Australia isolate [26].

### Phylogenetic analyses

Amino acid sequences inferred from the 12 protein-coding genes of the two *Chabertia* spp. worms were concatenated into a single alignment, and then aligned with those of 14 other Strongylida nematodes (*Angiostrongylus cantonensis*, GenBank accession number NC_013065 [30]; *Angiostrongylus costaricensis*, NC_013067 [30]; *Angiostrongylus vasorum*, JX268542 [31]; *Aelurostrongylus abstrusus*, NC_019571 [32]; *Chabertia ovina* Australia isolate, NC_013831 [26]; *Cylicocyclus insignis*, NC_013808 [26]; *Metastrongylus pudendotectus*, NC_013813 [26]; *Metastrongylus salmi*, NC_013815 [26]; *Oesophagostomum dentatum*, FM161882 [17]; *Oesophagostomum quadrispinulatum*, NC_014181 [17]; *Oesophagostomum asperum*, KC715826 [33]; *Oesophagostomum columbianum*, KC715827 [33]; *Strongylus vulgaris*, NC_013818 [26]; *Syngamus trachea*, NC_013821 [26], using the Ancylostomatoidea nematode, *Necator americanus*, NC_003416 as the outgroup [29]. Any regions of ambiguous alignment were excluded using Gblocks (http://molevol.cmima.csic.es/castresana/Gblocks_server.html) [34] with the default parameters (Gblocks removed 1.6% of the amino acid alignments) and then subjected to phylogenetic analysis using Bayesian Inference (BI) as described previously [35,36]. Phylograms were drawn using the program Tree View v.1.65 [37].

## Results

### Nuclear ribosomal DNA regions of the two *Chabertia* species

The rDNA region including ITS-1, 5.8S rDNA and ITS-2 were amplified and sequenced from *C. ovina* China isolates, and they were 852-854 bp (GenBank accession nos. KF913466-KF913471) in length, which contained 367-369 bp (ITS-1), 153 bp (5.8S rDNA) and 231-239 bp (ITS-2). These sequences were 862-866 bp in length for *C. erschowi* samples (GenBank accession nos. KF913448-KF913456), containing 375-378 bp (ITS-1), 153 bp (5.8S rDNA) and 239-245 bp (ITS-2).

### Features of the mt genomes of the two *Chabertia* species

**Figure 1:**
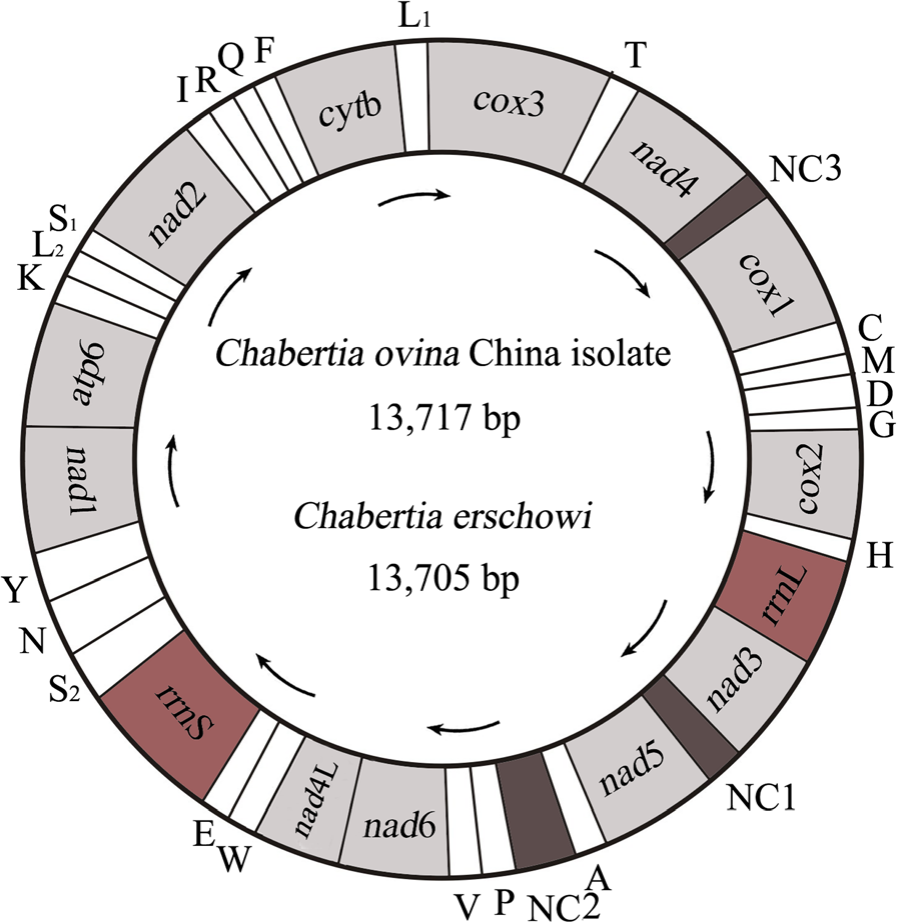
**Structure of the mitochondrial genomes for *Chabertia.*** Genes are designated according to standard nomenclature, except for the 22 tRNA genes, which are designated using one-letter amino acid codes, with numerals differentiating each of the two leucine- and serine-specifying tRNAs (L_1_ and L_2_ for codon families CUN and UUR, respectively; S_1_ and S_2_ for codon families AGN and UCN, respectively). “NCR-1, NCR-2 and NCR-3” refer to three non-coding regions.

The complete mt genome sequence of *C. ovina* China isolate and *C. erschowi* were 13,717 bp and 13,705 bp in length, respectively (GenBank accession nos. KF660604 and KF660603, respectively). The two mt genomes contain 12 protein-coding genes (*cox*1-3, *nad*1-6, *nad*4L, *cyt*b, *atp*6), 22 transfer RNA genes and two ribosomal RNA genes (*rrn*S and *rrn*L) (Table 2), but the *atp*8 gene is missing (Figure 1). The protein-coding genes are transcribed in the same directions, as reported for *Oesophagostomum* spp. [17,33]. Twenty-two tRNA genes were predicted from the mt genomes, which varied from 55 to 63 bp in size. The two ribosomal RNA genes (*rrn*L and *rrn*S) were inferred; *rrn*L is located between tRNA-His and *nad*3, and *rrn*S is located between tRNA-Glu and tRNA-Ser ^(UCN)^. Three AT-rich non-coding regions (NCRs) were inferred in the mt genomes (Table 2). For these genomes, the longest NCR (designated NC2; 250 bp for *C. ovina* China isolate and 240 bp for *C. erschowi* in length) is located between the tRNA-Ala and tRNA-Pro (Figure 1), have an A + T content of 83.75% and 84%, respectively.

**Table 2:** **Mitochondrial genome organization of *Chabertia erschowi *** **(CE) and *Chabertia ovina *** **China isolate (COC) and Australia isolate (COA)**

**Gene and region**	**Positions and nt sequence lengths (bp)**			**Initiation/termination codons**		
	**CE**	**COC**	**COA**	**CE**	**COC**	**COA**
*cox*1	2-1579 (1578)	2-1579 (1578)	2-1579 (1578)	ATT/TAA	ATT/TAA	ATT/TAA
tRN A-Cys (C)	1583-1637 (55)	1583-1639 (57)	1583-1639 (57)			
tRNA-Met (M)	1639-1697 (59)	1640-1699 (60)	1640-1699 (60)			
tRNA-Asp (D)	1699-1758 (60)	1700-1758 (59)	1699-1759 (61)			
tRNA-Gly (G)	1760-1816 (57)	1759-1814 (56)	1757-1814 (58)			
*cox*2	1817-2512 (696)	1815-2510 (696)	1814-2509 (696)	ATT/TAA	ATA/TAA	ATA/TAA
tRNA-His (H)	2512-2566 (55)	2512-2568 (57)	2511-2567 (57)			
*rrn*L	2573-3542 (970)	2572-3533 (962)	2570-3531 (962)			
*nad*3	3543-3881 (339)	3534-3869 (327)	3532-3867 (336)	ATT/TAA	ATT/TAA	ATT/TAA
Non-coding region (NC1)	3882-3965 (84)	3870-3949 (80)	3868-3947 (80)			
*nad*5	3967-5548 (1582)	3950-5531 (1582)	3948-5529 (1582)	ATT/ TAA	ATT/T	ATT/TAT
tRNA-Ala (A)	5549-5603 (55)	5532-5588 (57)	5530-5586 (57)			
Non-coding region (NC2)	5604-5853 (250)	5589-5828 (240)	5587-5825 (239)			
tRNA-Pro (P)	5854-5909 (56)	5829-5882 (54)	5826-5880 (55)			
tRNA-Val (V)	5927-5982 (56)	5930-5984 (55)	5914-5970 (57)			
*nad*6	5983-6417 (435)	5985-6416 (432)	5970-6401 (432)	ATA/ TAA	TTG/TAA	TTG/TAA
*nad*4L	6420-6653 (234)	6418-6651 (234)	6402-6635 (234)	ATT/ TAA	ATT/TAG	ATT/TAG
tRNA-Trp (W)	6681-6736 (56)	6655-6712 (58)	6639-6697 (59)			
tRNA-Glu (E)	6739-6794 (56)	6740-6797 (58)	6725-6784 (60)			
*rrn*S	6797-7492 (696)	6798-7493 (696)	6780-7479 (700)			
tRNA-Ser UCN (S2)	7493-7547 (55)	7494-7548 (55)	7480-7536 (57)			
tRNA-Asn (N)	7547-7603 (57)	7548-7605 (58)	7535-7593 (59)			
tRNA-Tyr (Y)	7610-7666 (57)	7608-7664 (57)	7595-7652 (58)			
*nad*1	7667-8539 (873)	7665-8537 (873)	7652-8524 (873)	ATT/TAA	ATT/TAA	ATT/TAA
*atp*6	8539-9138 (600)	8538-9137 (600)	8525-9121 (597)	ATT/TAA	ATT/TAA	ATT/TAG
tRNA-Lys (K)	9150-9211 (62)	9144-9206 (63)	9128-9191 (64)			
tRNA-LeuUUR (L_2_)	9222-9276 (55)	9215-9269 (55)	9197-9252 (56)			
tRNA-Ser AGN (S1)	9277-9335 (59)	9270-9327 (58)	9252-9304 (53)			
*nad*2	9336-10175 (840)	9328-10167 (840)	9308-10147 (840)	GTT/TAA	ATT/TAA	ATA/TAA
tRNA-Ile (I)	10176-10234 (59)	10175-10235 (61)	10151-10211 (61)			
tRNA-Arg (R)	10235-10289 (55)	10240-10294 (55)	10215-10270 (56)			
tRNA-Gln (Q)	10290-10345 (56)	10299-10353 (55)	10271-10326 (56)			
tRNA-Phe (F)	10346-10403 (58)	10354-10412 (59)	10326-10385 (60)			
*cyt*b	10404- 11516 (1113)	10413-11525 (1113)	10385-11497 (1113)	ATT/TAG	ATT/TAA	ATT/TAA
tRNA-Leu CUN (L1)	11517-11572 (56)	11529-11584 (56)	11501-11562 (62)			
*cox*3	11573-12338 (766)	11585-12350 (766)	11557-12327 (771)	ATT/T	ATT/T	ATT/TAA
tRNA-Thr (T)	12339-12397 (59)	12351-12404 (54)	12323-12377 (55)			
*nad*4	12398-13627 (1230)	12405-13634 (1230)	12376-13608 (1233)	TTG/TAA	TTG/TAA	ATT/TAA
Non-coding region (NC3)	13628 – 1 (75)	13635-1 (84)	13609-1 (75)			

### Comparative analyses between *C. ovina* and *C. erschowi*

The mt genome sequence of *C. erschowi* was 13,705 bp in length, 12 bp shorter than that of *C. ovina* China isolate, and 23 bp longer than that of *C. ovina* Australia isolate. The arrangement of the mt genes (i.e., 13 protein genes, 2 *rrn* genes and 22 tRNA genes) and NCRs were the same. A comparison of the nucleotide sequences of each mt gene as well as the amino acid sequences conceptually translated from individual protein-coding genes of the two *Chabertia* are given in Table 3. The greatest nucleotide variation between the *C. ovina* China isolate and *C. erschowi* was in the *nad*2 gene (19.4% and 17.92%), whereas least differences (7.33%) were detected in the *rrn*S gene, respectively (Table 3). The nucleotide sequence difference between the entire mt genome of *C. ovina* China isolate and that of *C. erschowi* was 15.33%. Sequence difference between the entire mt genome of *C. ovina* Australia isolate and that of *C. erschowi* was 15.48%. Sequence difference between the entire mt genome of *C. ovina* China isolate and that of *C. ovina* Australia isolate was 4.28%.

**Table 3:** **Nucleotide and/or predicted amino acid (aa) sequence differences for mt protein-coding and ribosomal RNA genes among *Chabertia erschowi *** **(CE) and *Chabertia ovina *** **China isolate (COC) and Australia isolate (COA)**

**Gene**	**Nucleotide length (bp)**			**Nucleotide difference (%)**			**Number of aa**			**aa difference (%)**		
	**CE**	**COC**	**COA**	**CE/COC**	**CE/COA**	**COC/COA**	**CE**	**COC**	**COA**	**CE/COC**	**CE/COA**	**COC/COA**
*atp*6	600	600	597	14.33	14.83	5.00	199	199	198	11.06	14.57	7.54
*nad*1	873	873	873	13.63	13.97	4.35	290	290	290	5.17	8.62	3.79
*nad*2	840	840	840	19.40	20.12	3.93	279	279	279	17.92	17.20	2.15
*nad*3	339	336	336	17.40	17.70	6.55	112	112	112	16.96	16.07	7.14
*nad*4	1230	1230	1233	17.64	18.82	5.43	409	409	410	14.67	17.80	4.88
*nad*4L	234	234	234	11.97	12.39	4.70	77	77	77	7.79	7.79	0
*nad*5	1582	1582	1582	17.51	17.32	4.87	527	527	527	14.42	13.66	2.47
*nad*6	435	432	432	19.08	19.31	5.56	144	143	143	15.97	17.36	1.40
*cox*1	1578	1578	1578	11.98	12.86	4.06	525	525	525	0.57	0.57	0
*cox*2	696	696	696	13.36	13.65	4.89	231	231	231	0.87	0.87	0
*cox*3	766	766	771	14.75	14.01	5.06	255	255	256	3.14	2.35	1.17
*cyt*b	1113	1113	1113	16.80	16.89	4.67	370	370	370	9.73	9.73	1.08
*rrn*S	696	696	700	7.33	7.71	1.86	-	-	-	-	-	-
*rrn*L	970	962	962	13.61	13.92	3.20	-	-	-	-	-	-

**Figure 2:**
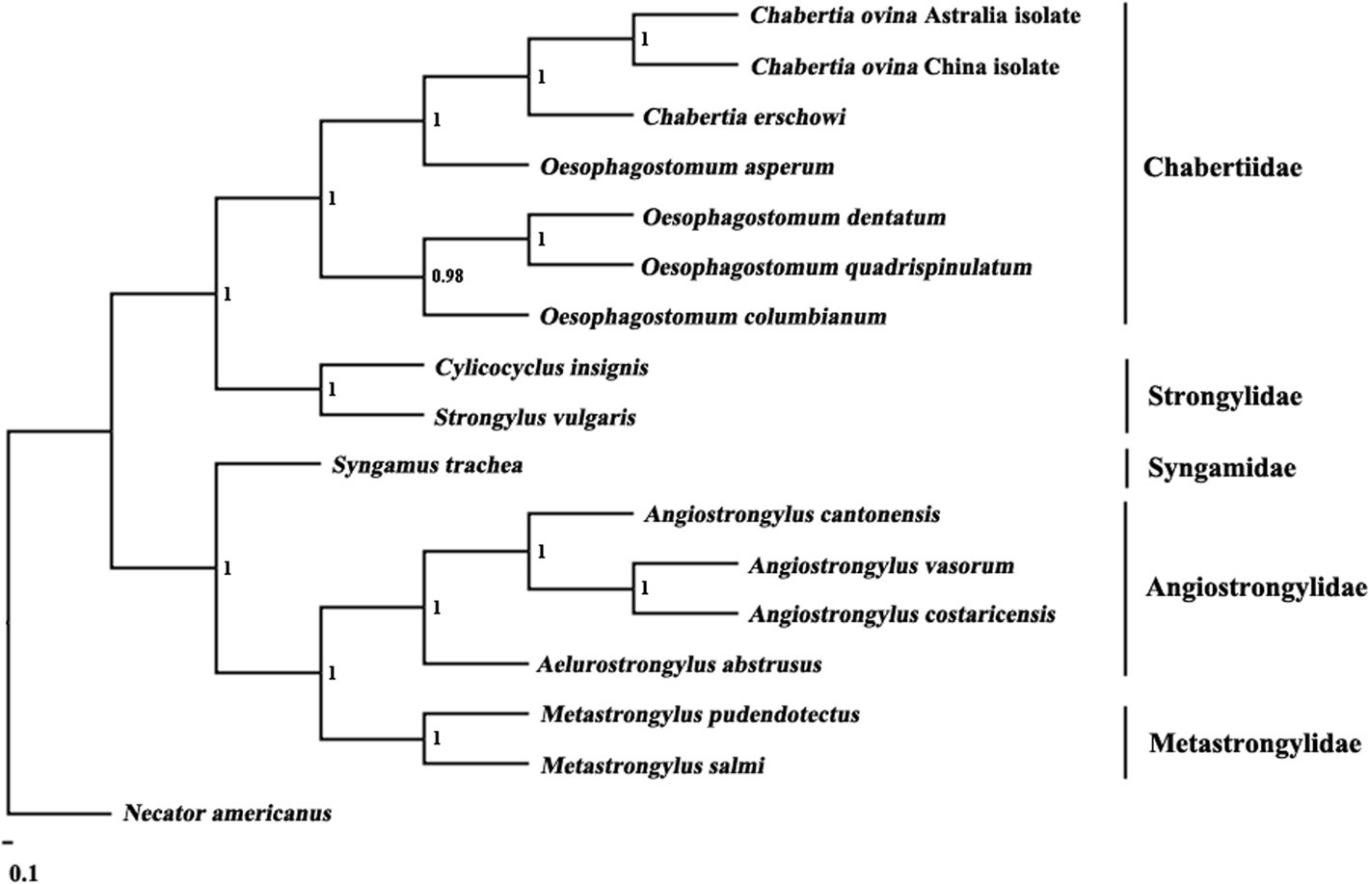
**Inferred phylogenetic position of *Chabertia *within Strongylida nematodes.** Analysis of the concatenated amino acid sequence data representing 12 protein-coding genes by Bayesian inference (BI), using *Necator americanus* (NC_003416) as the outgroup.

The difference in the concatenated amino acid sequences of the 12 protein-coding genes of the *C. ovina* China isolate and those of *C. erschowi* was 9.36%, 10% between those of the *C. ovina* Australia isolate and those of *C. erschowi*, and 2.37% between those of the *C. ovina* China isolate and those of *C. ovina* Australia isolate. The amino acid sequence differences between each of the 12 protein-coding genes of the *C. ovina* Australia isolate and the corresponding homologues of *C. erschowi* ranged from 0.57-17.92%, with COX1 being the most conserved and NAD2 the least conserved proteins (Table 3). Phylogenetic analyses of concatenated amino acid sequence data sets, using *N. americanus* as the outgroup, revealed that the *Chabertia* and *Oesophagostomum* were clustered together, with absolute support (posterior probability (pp) = 1.00) support (Figure 2).

**Figure 3:**
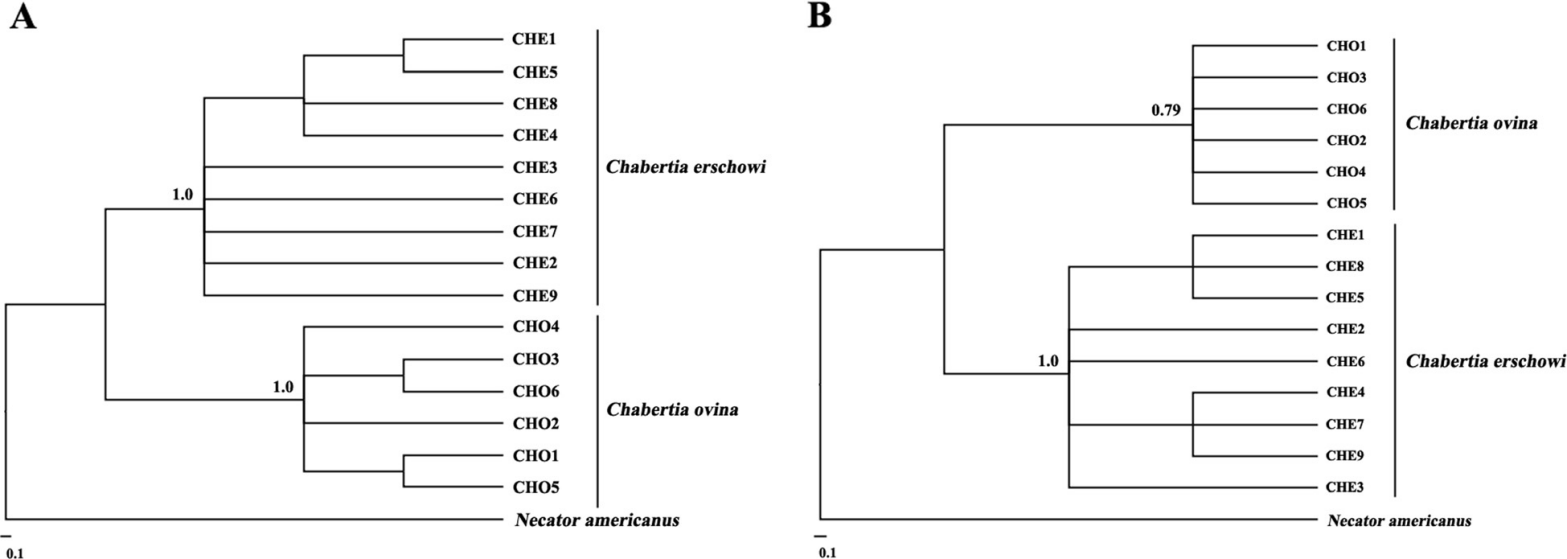
**Inferred genetic relationships of 15 individual *Chabertia *specimens.** The analyses were carried out by Bayesian inference (BI) based on mitochondrial *rrn*S **(A)** and *nad*2 **(B)** sequence data, using *Necator americanus* as the outgroup.

Sequence variation in complete *nad*2 gene was assessed among 15 individuals of *Chabertia* from goats and yaks. Sequences of the six *C. ovina* China isolate individuals were the same in length (840 bp) (GenBank accession nos. KF913472-KF913477). Nucleotide variation among the six *C. ovina* China isolate individuals was detected at 18 sites (18/840; 2.1%). Sequences of the nine *C. erschowi* individuals were the same in length (840 bp) (GenBank accession nos. KF913484-KF913492). Nucleotide variation also occurred at 23 sites (23/840; 2.7%). All 15 alignments of the *nad*2 sequences revealed that all individuals of *Chabertia* differed at 182 nucleotide positions (182/840; 21.7%). Phylogenetic analysis of the *nad*2 sequence data revealed strong support for the separation of *C. ovina* and *C. erschowi* individuals into two distinct clades (Figure 3A).

Sequence variation in complete *rrn*S gene was assessed among 15 individuals of *Chabertia* from goat and yak. Sequences of the *rrn*S gene from the six *C. ovina* China isolate individuals were the same in length (696 bp) (GenBank accession nos. KF913478-KF913483). Nucleotide variation among the six *C. ovina* China isolate individuals was detected at seven sites (7/696; 1.0%). Sequences of the *rrn*S gene from the nine *C. erschowi* individuals were the same in length (696 bp) (GenBank accession nos. KF913457-KF913465). Nucleotide variation also occurred at 6 sites (6/696; 0.9%). All 15 alignments of the *rrn*S sequences revealed that all individuals of *Chabertia* differed at 56 nucleotide positions (56/696; 8.05%). Phylogenetic analysis of the *rrn*S sequence data revealed strong support for the separation of *C. ovina* and *C. erschowi* individuals into two distinct clades (Figure 3B).

The ITS-1 and ITS-2 sequences from 10 individual adults of *C. ovina* China isolate were compared with that of 6 individual adults of *C. erschowi.* Sequence variations were 0–2.9% (ITS-1) and 0–2.7% (ITS-2) within the two *Chabertia* species, respectively. However, the sequence differences were 6.3-8.2% (ITS-1) and 10.4-13.6% (ITS-2) between the *C. ovina* China isolate and *C. erschowi*.

## Discussion

*Chabertia* spp. is responsible for economic losses to the livestock industries globally. Although several *Chabertia* species have been described from various hosts based on the microscopic features of the adult worms (e.g. cervical groove and cephalic vesicle), it is not clear whether *C. erschowi* is valid as a separate species due to unreliable morphological criteria. For this reason, we employed a molecular approach, so that comparative genetic analyses could be conducted.

In the present study, substantial levels of nucleotide differences (15.33%) were detected in the complete mt genome between *C. ovina* China isolate and *C. erschowi*, and 15.48% between *C. ovina* Australia isolate and *C. erschowi*. These mtDNA data provide strong support that *C. erschowi* represents a single species because a previous comparative study has clearly indicated that variation in mtDNA sequences between closely-related species were typically 10%-20% [13].

The difference in amino acid sequences of the concatenated 12 proteins encoded by the complete mt genome between *C. ovina* China isolate and *C. erschowi* is 9.36%, and 10% between the *C. ovina* Australia isolate and *C. erschowi*. This level of amino acid variation is higher than those of other nematodes. Previous studies of other congener nematodes have detected low level differences in 12 protein sequences. For example, differences in amino acid sequences between *A. duodenale* and *A. caninum* is 4.1% [29,38], and between *Toxocara malaysiensis* and *Toxocara cati* is 5.6% [39], and between *O. dentatum* and *O. quadrispinulatum* is 3.22% [17]. In addition, substantial levels of nucleotide differences (6.3%-8.2% in ITS-1 and 10.4-13.6% in ITS-2) were also detected between *C. ovina* China isolate and *C. erschowi*. These results also indicate that *C. erschowi* is a separate species from *C. ovina*. This proposal was further supported by phylogenetic analysis based on mtDNA sequences (Figure 3), although, to date, only small numbers of adult worms have been studied molecularly. Clearly, larger population genetic and molecular epidemiological studies should be conducted using the mt and nuclear markers defined in this study to further test this proposal/hypothesis.

## Conclusion

The findings of this study provide robust genetic evidence that *C. erschowi* is a separate and valid species from *C. ovina*. The mtDNA and rDNA datasets reported in the present study should provide useful novel markers for further studies of the taxonomy and systematics of *Chabertia* spp. from different hosts and geographical regions.

## Competing interests

The authors declare that they have no competing interests.

## Authors’ contributions

XQZ and GHL conceived and designed the study, and critically revised the manuscript. GHL, LZ and HQS performed the experiments, analyzed the data and drafted the manuscript. GHZ, JZC and QZ helped in study design, study implementation and manuscript revision. All authors read and approved the final manuscript.
